# Supplementation with Mo, Co, and Ni Enhances the Effectiveness of Co-Inoculation with the Rhizobacteria *Azospirillum brasilense* and *Bradyrhizobium diazoefficiens* in Soybean

**DOI:** 10.3390/microorganisms13122680

**Published:** 2025-11-25

**Authors:** Mateus Neri Oliveira Reis, Luciana Cristina Vitorino, Marialva Alvarenga Moreira, Alex Santos Macedo, Letícia Ferreira de Sousa, Lucas Loram Lourenço, Layara Alexandre Bessa

**Affiliations:** 1 Laboratory of Metabolism and Genetics of Biodiversity, Instituto Federal Goiano, Rio Verde Campus, Highway Sul Goiana, Km 01, Rio Verde 75901-970, GO, Brazil; mateusnerioliveira@hotmail.com (M.N.O.R.); alex.macedo@estudante.ifgoiano.edu.br (A.S.M.); leticia.sousa@estudante.ifgoiano.edu.br (L.F.d.S.); lucas.loram@outlook.com (L.L.L.); layara.bessa@ifgoiano.edu.br (L.A.B.); 2 Laboratory of Agricultural Microbiology, Instituto Federal Goiano, Rio Verde Campus, Rio Verde 75901-970, GO, Brazil; 3 Empresa de Pesquisa Agropecuária de Minas Gerais—EPAMIG Sudeste, Campo Experimental Vale do Piranga, Highway Luiz Martins Soares, Km 05, Zona Rural, P.O. Box 01, Oratórios 35439-000, MG, Brazil; marialva.moreira@epamig.br

**Keywords:** biological nitrogen fixation, diazotrophic rhizobacteria, bioinoculant, plant growth promotion

## Abstract

Efficient biological nitrogen fixation (BNF) is crucial for sustainable soybean productivity. Current strategies involve the use of *Bradyrhizobium diazoefficiens* and co-inoculation with plant growth-promoting bacteria like *Azospirillum brasilense*. To further optimize BNF and plant performance, we investigated the effect of co-inoculation with *A. brasilense* and *B. diazoefficiens* combined with the strategic application of the micronutrients Molybdenum (Mo), Cobalt (Co), and Nickel (Ni) on soybean grown under greenhouse conditions. We evaluated plant growth, photosynthetic parameters, accumulation of N, nitrate reductase activity, and *nifH* gene expression at the R1 reproductive stage. Our main finding was that the co-inoculation combined with the simultaneous application of Mo, Co, and Ni significantly maximized vegetative growth, photochemical efficiency, and BNF. Specifically, this triple supplementation increased *nifH* gene expression (0.22) compared to the inoculated control (0.003), leading to a substantial enhancement of photosynthetic parameters, including photosystem II (PSII) efficiency and net carbon assimilation (*A*). For example, the total dry mass was 14.36 g in the Mo + Co + Ni + AZO + BRADY combination and 6.50 g in the non-inoculated and non-micronutrient-treated plants. The total N content was also higher in the plants treated with Mo + Co + Ni + AZO + BRADY (73.20 g kg^−1^). Crucially, the data also demonstrated that excessive levels of Co impaired the symbiosis, underscoring the necessity of precise dose management. These results confirm the strong synergistic potential of combining microbial co-inoculation with targeted mineral nutrition as a high-impact, sustainable strategy for boosting soybean productivity.

## 1. Introduction

Sustainable agricultural practices, such as the use of nitrogen-fixing microorganisms, have been widely adopted worldwide as alternatives to reduce the reliance on synthetic nitrogen fertilizers [1,2]. Diazotrophic strains selected for their high efficiency in converting atmospheric nitrogen to NH_3_ have become indispensable in the context of biotechnological advancements in agriculture. In the realm of biological nitrogen fixation (BNF), most commercial biofertilizers are based on *Azospirillum brasilense* and *Bradyrhizobium* spp. [3,4]. These microorganisms colonize the roots of major crops, such as soybeans, enhancing development, growth, and productivity [5,6,7].

Soybean establishes symbiosis with *Bradyrhizobium* and *Azospirillum* because these bacteria express genes of the *nif* complex, which are essential for BNF [8,9]. Studies indicate that co-inoculation of these bacteria is an effective strategy for improving crop productivity [10]. Furthermore, co-inoculation in combination with micronutrients has been suggested as an important management practice to increase soybean yield [11]. Given that molybdenum (Mo), cobalt (Co), and nickel (Ni) are critical components of the symbiotic process, necessary for activation or synthesis of BNF-related enzymes [12,13,14], we hypothesized that co-inoculation of soybean with *A. brasilense* and *B. diazoefficiens* in combination with Mo, Co, and Ni could enhance plant physiological performance, resulting in improved growth.

In soybeans, diazotrophic bacteria act either in the nodules (*Bradyrhizobium*) or in the rhizosphere (*Azospirillum*) [3]. Within nodules, Co is involved in cobalamin-dependent synthesis of leghemoglobin [15]. Meanwhile, diazotrophic bacteria utilize Mo, iron (Fe), ATP, and protons (H^+^) to convert atmospheric N_2_ into ammonia via nitrogenase, which cleaves the N≡N triple bond to generate NH_3_ and H_2_ [12,16,17,18]. Ni serves as a cofactor in hydrogenase, which recycles H_2_ produced during nitrogenase activity; excess H_2_ can compete with N_2_ at the nitrogenase active site, potentially inhibiting BNF [13,19].

The NH_3_ produced is protonated to NH_4_^+^ and subsequently converted into ureides (allantoin and allantoic acid), which are transported via the xylem to the leaves [20,21,22]. In the leaves, ureides are catabolized through the purine degradation pathway into urea, which is hydrolyzed by urease, a Ni-dependent enzyme, producing NH_3_ and CO_2_ that are incorporated into amino acids and proteins [23,24].

Molybdenum (Mo) also acts as a component of several essential enzymes for functional and metabolic processes, such as xanthine oxidoreductase, aldehyde oxidase, and sulfite oxidase [25,26]. Mo homeostasis in plant cells is strictly controlled, suggesting a vital role in plant adaptation to local environments [27]. Additionally, cobalamin-dependent enzymes have been observed in plants, such as peroxidases, leucine 2,3-aminomutase, and methylmalonyl-CoA mutase, reinforcing the relevance of Co [28].

Thus, studies show that the micronutrients Co, Mo, and Ni are important in biochemical processes that extend beyond BNF. The management of Ni in agricultural systems, in turn, has emerged as a promising and low-cost strategy to enhance productivity and sustainability in soybean cultivation by improving nitrogen metabolism through enzymes like urease and NiFe-hydrogenase and optimizing calcium uptake as well as the efficiency of water and nutrient use by plants [29]. This micronutrient focus is crucial because soybean is a legume that produces high-protein grains, making it vital in combating global food insecurity [30]. Soybean is the world’s leading oilseed crop and ranks among the top five food crops globally [31]. Currently, soybeans are cultivated across $122.10 million hectares, yielding an annual production of $353 million metric tons (MT) and boasting a productivity rate of $28.00 quintals per hectare, with Brazil and the USA as the leading producers [32]. Nevertheless, its high yield is intrinsically linked to an adequate N supply [33], which motivates the continuous search for new technologies that stimulate BNF in the nodules or rhizosphere.

Recent studies have demonstrated that the co-inoculation of soybean with *A. brasilense* and *B. diazoefficiens*, when associated with the supply of Mo and Co and the use of cell protectants, can result in a greater number of pods per plant [34]. However, despite these benefits, the micronutrients also have a flip side. The work by Silveira et al. [35] showed that high doses of Mo and Co applied to soybeans can be toxic to the *Bradyrhizobium* genus, negatively impacting the crop’s agronomic characteristics. Conversely, the study by Barbosa et al. [11] indicated that the foliar application of *A. brasilense* and *B. diazoefficiens* co-inoculation increased soybean yield by 10% in plants fertilized with Co and Mo. In this context, the present study aims to evaluate the effects of different formulations containing Mo, Co, and Ni in combination with *A. brasilense* and *B. diazoefficiens* on soybean growth, nodulation, physiological performance, tissue nitrogen accumulation, and BNF activity via *nifH* gene expression in nodules. The study seeks to provide an alternative to conventional nitrogen fertilization, promoting improved agricultural management and production efficiency.

## 2. Materials and Methods

### 2.1. Plant Material, Inoculants, and Experimental Conditions

The experiment was conducted at the Plant Biotechnology Complex of the Instituto Federal Goiano–Campus Rio Verde, located at 17°48′15.9″ S and 50°54′19.5″ W. A randomized complete block design was employed, with treatments applied in 3 L pots and five replicates per treatment. Each pot contained two plants, which together constituted a single experimental unit. Soybean seeds of the cultivar Bônus 8579 RSF IPRO were used. Prior to planting, the soil was analyzed for chemical and nutritional properties (Table 1), and soil acidity was corrected with 2000 kg ha^−1^ of dolomitic limestone (28% CaO + 14.5% MgO).

The bacterial strains used in this experiment were obtained from the Simple Verde^®^ strain bank (Simple Agro Corporation, Rio Verde, GO, Brazil) and provided at an initial concentration of 10^8^ CFU mL^−1^. The inoculants and micronutrients were applied in the planting furrow, just above the seed. The seeds were de-infested superficially for the removal of the epiphytes through repeated rinses under running water, followed by agitation in water and neutral detergent at 70 rpm for 10 min. After successive rinses, the seeds were immersed in 70% ethanol for 1 min, sodium hypochlorite (2.5% of active chlorine) for 5 min, and then 70% ethanol for 30 s, followed by three rinses with autoclaved distilled water. Subsequently, soybean seeds were sown in furrows positioned at the center of each pot, and the recommended volumes of biological inoculants were applied using a volumetric pipette: 200 mL ha^−1^ for *A. brasilense* and 200 mL ha^−1^ for *B. diazoefficiens*. In the co-inoculation treatments, the same volumes were maintained for each rhizobacterium. Micronutrients were applied in different combinations (Table 2) using a volumetric pipette: 30 g ha^−1^ of ammonium molybdate (Mo), 3 g ha^−1^ of cobalt sulfate (Co), and 30 g ha^−1^ of nickel sulfate (Ni). To mitigate the risk of toxicity, we opted to use Mo and Co doses corresponding to approximately 60% of the recommendation established by the Comissão de Química e Fertilidade do Solo (CQFS) [36]. These doses were defined based on the quantities of ammonium molybdate and cobalt sulfate tested, respectively, in the studies by Picazeviz et al. [37] and Marcondes and Caires [38]. Regarding Ni, the dose used was determined based on the work by Lavres et al. [19]. Control plants received no micronutrients, with or without rhizobacterial inoculation. All pots were maintained in a greenhouse, monitored daily, and watered according to plant requirements. All biometric, physiological, and nitrogen content analyses were performed on five replicates of each treatment, with each replicate consisting of two plants per pot.

### 2.2. Biometric Evaluations and Nodulation

Data were collected for plant height (cm), stem diameter (cm), shoot dry mass (g), root dry mass (g), total dry mass (g), and nodule number. Evaluations were performed at the R1 phenological stage, corresponding to the period of maximum nodulation in soybean. For nodulation assessment, roots were carefully washed under running water, and nodules were counted per plant. Shoots were separated from roots, and both plant parts were placed in paper bags and dried in a forced-air oven at 65 °C until constant weight was achieved. Subsequently, shoot and root dry mass were measured using a precision balance, and results were expressed as g plant^−1^.

### 2.3. Physiological Assessments

All physiological evaluations were performed at the R1 stage, using the third fully expanded leaf counted from the apical meristem toward the base of the plant. Photosynthetic pigment content was assessed with a portable chlorophyll meter (ClorofiLOG CFL1030, Falker^®^, Porto Alegre, RS, Brazil), determining chlorophyll *a*, chlorophyll *b*, and total chlorophyll indices, expressed as the Falker chlorophyll index (FCI).

Transient chlorophyll a fluorescence (OJIP) was measured using a portable FluorPen FP 100 fluorometer (Photon Systems Instruments, Drasov, Czech Republic). Leaves were dark-adapted for 30 min to ensure complete oxidation of the photosynthetic electron transport system. Subsequently, leaves were exposed to a 3000 µmol m^−2^ s^−1^ pulse of blue light, and fluorescence was recorded at: F_0_ (50 µs, all PSII reaction centers open, O-step), J (2 ms), I (30 ms), and F_M_ (all PSII reaction centers closed, P-step). These measurements were used to calculate several PSII bioenergetic parameters following Strasser et al. [39], including: specific flux of light absorption per reaction center (ABS/RC); energy flux trapped per reaction center at t = 0 (TR_0_/RC); electron transport flux per reaction center (ET_0_/RC); specific flux of energy dissipation at the antenna chlorophylls (DI_0_/RC); maximum PSII quantum efficiency (F_V_/F_M_); and the photosynthetic performance index (PI_Abs), reflecting the overall energy conversion from light absorption to plastoquinone reduction.

Gas exchange measurements were performed between 07:00 and 10:00 a.m. using an infrared gas analyzer coupled with a fluorometer (LI-6800xt, LI-COR Inc., Lincoln, NE, USA). The following parameters were determined: net carbon assimilation rate (*A*, µmol CO_2_ m^−2^ s^−1^), transpiration rate (*E*, mmol H_2_O m^−2^ s^−1^), intercellular CO_2_ concentration (*Ci*, µmol m^−2^ s^−1^), and stomatal conductance (*Gsw*, mol H_2_O m^−2^ s^−1^).

### 2.4. N Content and Nitrate Reductase (NR, EC 1.6.6.1) Activity

At the R1 growth stage, soybean plants were sampled for nitrogen content analysis. Shoots (leaves and stems) and roots were separated and dried in a forced-air oven at 65 °C until constant weight. The dried material was then ground using a Willey-type mill (R-TE-650/1, Tecnal, Piracicaba, SP, Brazil). Total nitrogen content was determined following the methodology described by Malavolta et al. [40].

Leaf samples were also collected at R1 and immediately stored at −80 °C for the determination of nitrate reductase (NR) activity, according to the procedure described by Hageman and Reed [41]. For enzyme extraction, 0.2 g of fresh leaf tissue was homogenized with 2 mL of extraction buffer composed of potassium phosphate buffer (pH 7.5), 30 nM KNO_3_, and 2% (*v*/*v*) propanol. The samples were vacuum-infiltrated for 5 min, and a 1 mL aliquot was taken to measure the initial nitrite concentration. Another 1 mL aliquot was incubated in a water bath at 31 °C for 1 h. After incubation, the samples were filtered, and the reaction mixture was prepared by adding 1 mL of the filtrate to a colorimetric reagent containing 1% sulfanilamide and 0.02% N-(1-naphthyl)ethylenediamine dihydrochloride, prepared in 1.5 M and 0.2 M HCl, respectively. The mixture was incubated at room temperature for 30 min, and absorbance was measured at 540 nm using a UV-Vis spectrophotometer (UV-1800, Shimadzu, Barueri, SP, Brazil). Nitrate reductase activity was quantified against a standard curve prepared with NO_2_^−^ (0–20 µg mL^−1^) and expressed as µg NO_2_^−^ h^−1^ mg^−1^ fresh weight (FW).

### 2.5. nifH Gene Expression Levels

Total RNA was extracted from individual root nodule samples of each treatment using the RNeasy Mini Kit (Qiagen, Hilden, North Rhine-Westphalia, Germany), following the manufacturer’s protocol. First-strand cDNA was synthesized from 10 µL of DNA-free RNA in a 20 µL reaction volume using the Power SYBR Green RT-PCR Reverse Transcription Kit (Applied Biosystems, Waltham, MA, USA). The resulting cDNA was subsequently used as a template for quantitative RT-PCR analyses. For amplification of the target gene *nifH*, reactions were performed using SYBR Green PCR Master Mix (Applied Biosystems, USA) with gene-specific primers: forward 5′-CATACATCGCCATCATCTCG-3′ and reverse 5′-ATCAAGCTCGGCTACAAGGA-3′. Each 20 µL PCR reaction contained 10 µL of SYBR Green Master Mix, 0.6 µL of each primer, 1 µL of cDNA, and 7.8 µL of sterile water. Relative expression levels of *nifH* were normalized to the constitutive rec*A* gene.

### 2.6. Statistical Analyses

The data were subjected to one-way analysis of variance (ANOVA) to evaluate the effects of different combinations of Mo, Co, and Ni with the rhizobacteria *A. brasilense* (AZO) and *B. diazoefficiens* (BRADY), totaling 25 treatments. When significant differences were detected, means were compared using the Scott–Knott test (*p* < 0.05). Additionally, the individual effect of each treatment was compared with the overall control (uninoculated plants without micronutrient supplementation) using Dunnett’s test (*p* < 0.05). Multivariate patterns among variables were explored through Canonical Variable Analysis (CVA) using the multivariate analysis package [42] to identify the canonical axes explaining the total variance. Pearson’s correlation coefficients were calculated and visualized with the corrplot package [43]. All statistical analyses were performed using R software version 4.5.1 [44].

## 3. Results

**Figure 1:**
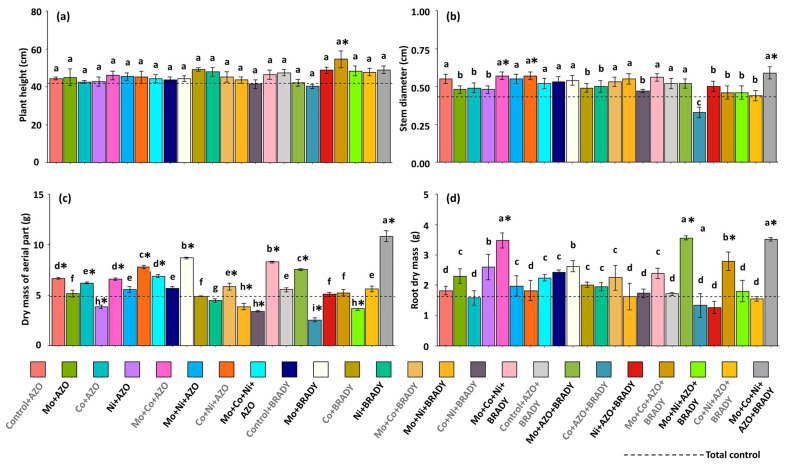
Growth of *Glycine max* L. plants subjected to different combinations of the micronutrients Mo, Co, and Ni and the diazotrophic rhizobacteria *Azospirillum brasilense* (AZO) and *Bradyrhizobium diazoefficiens* (BRADY). Plant height (**a**), stem diameter (**b**), dry mass of aerial part (**c**), and root dry mass (**d**). Bars followed by the same letter do not differ statistically according to the Scott–Knott test (*p* < 0.05). The dashed line represents the total control (absence of micronutrient treatment and microbial inoculation). Asterisks (*) indicate statistical differences from the total control based on the Dunnett test (*p* < 0.05).

Overall, soybean plant height was not significantly influenced by the nutritional and inoculation treatments. However, plants treated with the combination Mo + Co + AZO + BRADY exhibited greater average height (54.60 cm) compared to the total control (41.80 cm) (Figure 1a). Stem diameter, in contrast, was significantly affected by the treatments. Plants receiving Co + AZO + BRADY developed the smallest average diameters (0.33 cm), whereas those treated with Mo + Co + AZO, Co + Ni + AZO, and the complete formulation Mo + Co + Ni + AZO + BRADY showed greater stem diameters (0.57, 0.57, and 0.59 cm, respectively) than the total control (Figure 1b).

Shoot dry mass was highest in plants subjected to the complete treatment (Mo + Co + Ni + AZO + BRADY), with an average of 10.85 g per plant (Figure 1c). In contrast, plants from the total control accumulated the lowest shoot dry mass (4.87 g). The combination Co + AZO + BRADY also resulted in reduced biomass accumulation, with the lowest mean value observed (2.53 g). Root development, however, was enhanced in plants treated with Mo + Co + AZO, Mo + AZO + BRADY, and Mo + Co + Ni + AZO + BRADY, with average root dry masses of 3.48, 3.56, and 3.51 g, respectively, all of which were higher than that of the total control (1.63 g) (Figure 1d).

**Figure 2:**
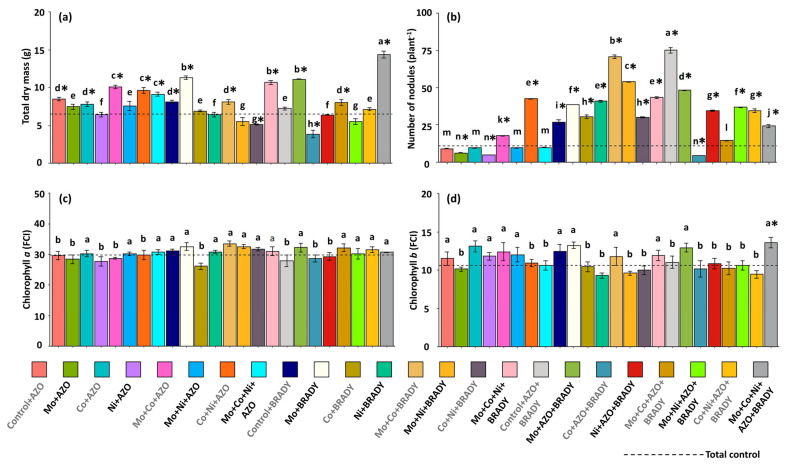
Growth, nodulation, and photosynthetic pigment accumulation in *Glycine max* L. plants subjected to different combinations of the micronutrients Mo, Co, and Ni and the diazotrophic rhizobacteria *Azospirillum brasilense* (AZO) and *Bradyrhizobium diazoefficiens* (BRADY). Total dry mass (**a**), number of nodules (**b**), chlorophyll *a* index (**c**), and chlorophyll *b* index (**d**). Bars followed by the same letter do not differ statistically according to the Scott–Knott test (*p* < 0.05). The dashed line represents the total control (absence of micronutrient treatment and microbial inoculation). Asterisks (*) indicate statistical differences from the total control based on the Dunnett test (*p* < 0.05).

The data on total dry mass corroborated the trends observed for shoot dry mass, with plants subjected to the complete treatment (Mo + Co + Ni + AZO + BRADY) accumulating the highest total biomass (14.36 g) (Figure 2a). This treatment was followed by Mo + BRADY, Mo + Co + Ni + BRADY, and Mo + AZO + BRADY, with mean total dry masses of 11.30, 10.68, and 11.10 g, respectively. These results suggest that the presence of *B. diazoefficiens* enhances the utilization of micronutrients by soybean plants. In general, plants from the total control exhibited lower total dry mass (6.50 g), whereas the lowest values were recorded for those treated with Co + AZO + BRADY (3.86 g). Regarding nodule formation, plants grown without micronutrient supplementation but co-inoculated with *A. brasilense* and *B. diazoefficiens* (Control + AZO + BRADY) produced the highest number of nodules (75), followed by those treated with Mo + Co + BRADY (71) (Figure 2b). The total control plants exhibited a reduced number of nodules (10), while the Co + AZO + BRADY treatment resulted in the lowest mean value (4.4).

The chlorophyll *a* index was not significantly affected by either the nutritional or inoculation treatments and did not differ from that of the total control (Figure 2c). However, the chlorophyll *b* index was significantly higher only in plants receiving the complete treatment (Mo + Co + Ni + AZO + BRADY), reaching 13.60 FCI (Figure 2d).

**Figure 3:**
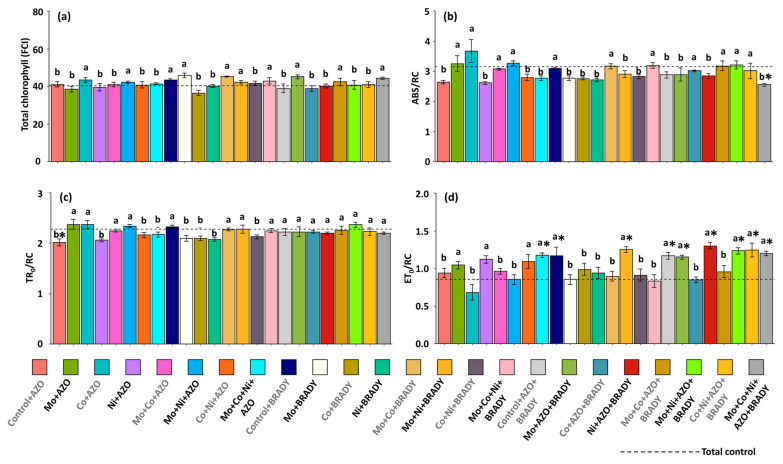
Total chlorophyll and chlorophyll *a* fluorescence parameters in leaves of *Glycine max* L. plants subjected to different combinations of the micronutrients Mo, Co, and Ni and the diazotrophic rhizobacteria *Azospirillum brasilense* (AZO) and *Bradyrhizobium diazoefficiens* (BRADY). Total chlorophyll (**a**), ABS/RC (**b**), TR_0_/RC (**c**), and ET_0_/RC (**d**). Bars followed by the same letter do not differ statistically according to the Scott–Knott test (*p* < 0.05). The dashed line represents the total control (absence of micronutrient treatment and microbial inoculation). Asterisks (*) indicate statistical differences from the total control based on the Dunnett test (*p* < 0.05).

The total chlorophyll index followed the same trend observed for chlorophyll *a*, showing no significant effect of treatments compared with the total control (Figure 3a). The chlorophyll *a* fluorescence parameters indicated that the average specific light absorption flux per reaction center (ABS/RC) was lower than that of the total control in plants receiving the complete treatment (2.55) (Figure 3b). Conversely, the average energy flux trapped per reaction center (TR_0_/RC) was reduced only in plants treated with Control + AZO (2.02) compared with the control (Figure 3c). In contrast, plants subjected to the complete treatment exhibited a higher electron transport flux per reaction center (ET_0_/RC) (1.20) relative to the total control (0.85) (Figure 3d). Similar responses were observed in plants treated with Mo + Co + Ni + AZO, Control + BRADY, Mo + Ni + BRADY, Control + AZO + BRADY, Mo + AZO + BRADY, Ni + AZO + BRADY, Mo + Ni + AZO + BRADY, and Co + Ni + AZO + BRADY. These findings suggest that inoculation of *Glycine max* with *B. diazoefficiens*, either alone or in combination with micronutrients or the rhizobacterium *A. brasilense*, can enhance the photochemical performance of soybean plants.

**Figure 4:**
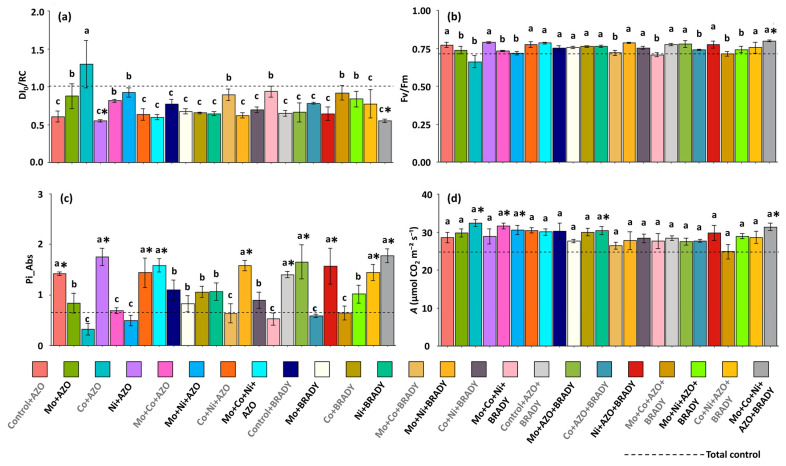
Chlorophyll *a* fluorescence parameters and net carbon assimilation rate in leaves of *Glycine max* L. plants subjected to different combinations of the micronutrients Mo, Co, and Ni and the diazotrophic rhizobacteria *Azospirillum brasilense* (AZO) and *Bradyrhizobium diazoefficiens* (BRADY). DI_0_/RC (**a**), Fv/Fm (**b**), Pi_Abs (**c**), and *A* (**d**). Bars followed by the same letter do not differ statistically according to the Scott–Knott test (*p* < 0.05). The dashed line represents the total control (absence of micronutrient treatment and microbial inoculation). Asterisks (*) indicate statistical differences from the total control based on the Dunnett test (*p* < 0.05).

The data on the specific energy dissipation flux at the chlorophyll level of the antenna complex (DI_0_/RC) reinforce the evidence that plants receiving the complete treatment (Mo + Co + Ni + AZO + BRADY) achieved greater photochemical stability, as indicated by the lowest average values for this parameter (0.55) (Figure 4a). Similar results were observed in plants treated with Ni + AZO. In contrast, plants treated with Co + AZO exhibited the highest averages (1.30), suggesting the occurrence of photochemical stress. The average ratio representing the maximum efficiency of PSII (Fv/Fm) was higher than and statistically different from that of the total control only in plants subjected to the complete treatment (0.80) (Figure 4b).

Overall, the photosynthetic performance index (PI_Abs) was low in plants from the total control (0.65), while higher values were observed in plants treated with the complete formulation (1.78), as well as in those subjected to Control + AZO, Ni + AZO, Co + Ni + AZO, Mo + Co + Ni + AZO, Mo + Ni + BRADY, Control + AZO + BRADY, Mo + AZO + BRADY, Ni + AZO + BRADY, and Co + Ni + AZO + BRADY (Figure 4c). Regarding the net carbon assimilation rate (A), plants under the complete treatment (31.37 µmol CO_2_ m^−2^ s^−1^) showed higher and significantly different averages compared with the total control (24.76 µmol CO_2_ m^−2^ s^−1^). Similar trends were also observed in plants treated with Co + AZO, Mo + Ni + AZO, Mo + Co + Ni + AZO, and Ni + BRADY.

**Figure 5:**
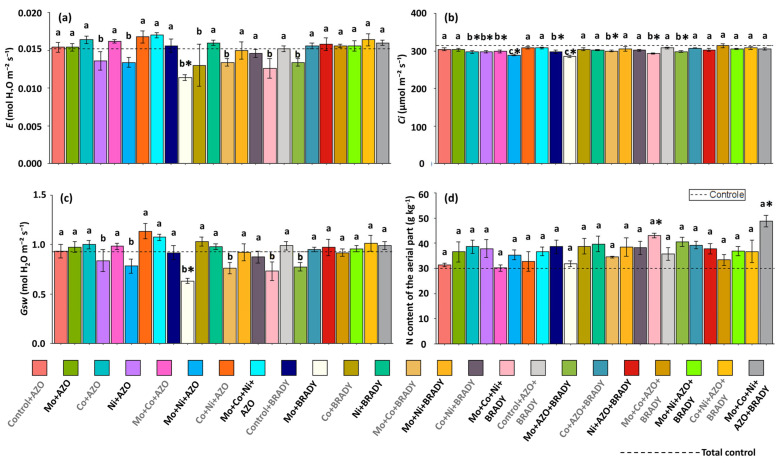
Gas exchange parameters and nitrogen content in leaves and shoots of *Glycine max* L. plants subjected to different combinations of the micronutrients Mo, Co, and Ni and the diazotrophic rhizobacteria *Azospirillum brasilense* (AZO) and *Bradyrhizobium diazoefficiens* (BRADY). *E* (**a**), *Ci* (**b**), *Gsw* (**c**), and shoot N content (**d**). Bars followed by the same letter do not differ statistically according to the Scott–Knott test (*p* < 0.05). The dashed line represents the total control (absence of micronutrient treatment and microbial inoculation). Asterisks (*) indicate statistical differences from the total control based on the Dunnett test (*p* < 0.05).

In general, inoculation and co-inoculation treatments did not differ significantly from the total control with respect to transpiration (*E*). Notably, very low transpiration rates were observed only in plants treated with Mo + BRADY (0.011 mmol H_2_O m^−2^ s^−1^) (Figure 5a). Conversely, internal CO_2_ concentration (*Ci*) tended to decrease in most treatments compared with the total control, with the lowest *Ci* values recorded in plants treated with Mo + Ni + AZO and Mo + BRADY (289.27 and 285.25 µmol CO_2_ m^−2^ s^−1^, respectively) (Figure 5b). As expected, stomatal conductance (*Gsw*) was reduced in treatments that showed low *E* and *Ci* values. Accordingly, plants subjected to Mo + BRADY exhibited the lowest average *Gsw* (0.63 mol H_2_O m^−2^ s^−1^) (Figure 5c).

The N content in the shoot was positively influenced by the treatment of soybean plants with the combination of the three evaluated micronutrients and *B. diazoefficiens* (Mo + Co + Ni + BRADY), as well as by the complete treatment, which also included *A. brasilense* (Mo + Co + Ni + AZO + BRADY). Plants subjected to these treatments showed higher average shoot N contents (43.10 and 48.73 g kg^−1^, respectively) compared to those grown without micronutrient application and without inoculation (29.97 g kg^−1^).

**Figure 6:**
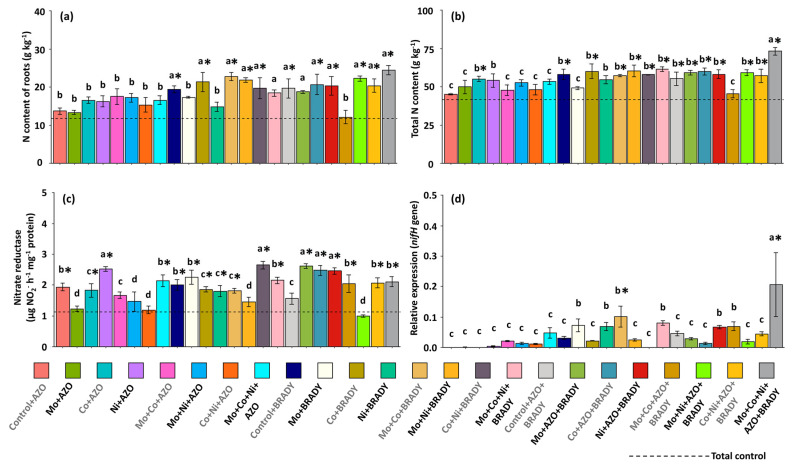
Nitrogen content, nitrate reductase activity, and relative expression of the *nifH* gene in *Glycine max* L. plants subjected to different combinations of the micronutrients Mo, Co, and Ni and the diazotrophic rhizobacteria *Azospirillum brasilense* (AZO) and *Bradyrhizobium diazoefficiens* (BRADY). Root N content (**a**), total N content (**b**), nitrate reductase activity (**c**), and *nifH* gene expression in nodules (**d**). Bars followed by the same letter do not differ statistically according to the Scott–Knott test (*p* < 0.05). The dashed line represents the total control (absence of micronutrient treatment and microbial inoculation). Asterisks (*) indicate statistical differences from the total control based on the Dunnett test (*p* < 0.05).

In the roots, the highest nitrogen contents were observed in most treatments that combined micronutrients with *B. diazoefficiens* and co-inoculation with *A. brasilense* (AZO + BRADY) (Figure 6a), reinforcing the importance of using *B. diazoefficiens* as an agronomic strategy to enhance N acquisition by *G. max*. The evaluation of total N content, however, demonstrated the superiority of the complete treatment in increasing N availability to the plants. The highest total N averages were recorded in plants under this treatment (73.20 g kg^−1^), which were significantly higher than those observed in the other treatments overall (Figure 6b).

Nitrate reductase activity in leaves was higher than that observed in the total control for most of the nutritional and inoculation treatments (Figure 6c), suggesting that different combinations of Mo, Co, and Ni can stimulate not only N assimilation but also N metabolism pathways in *G. max*. Regarding the relative expression of the *nifH* gene in nodules, activity was close to zero in the total control plants. In contrast, treatments containing BRADY, with or without AZO, tended to increase gene expression. The Mo + Co + BRADY treatment showed moderate expression levels (0.10), whereas the complete treatment resulted in the highest average *nifH* expression (0.22) (Figure 6d).

**Figure 7:**
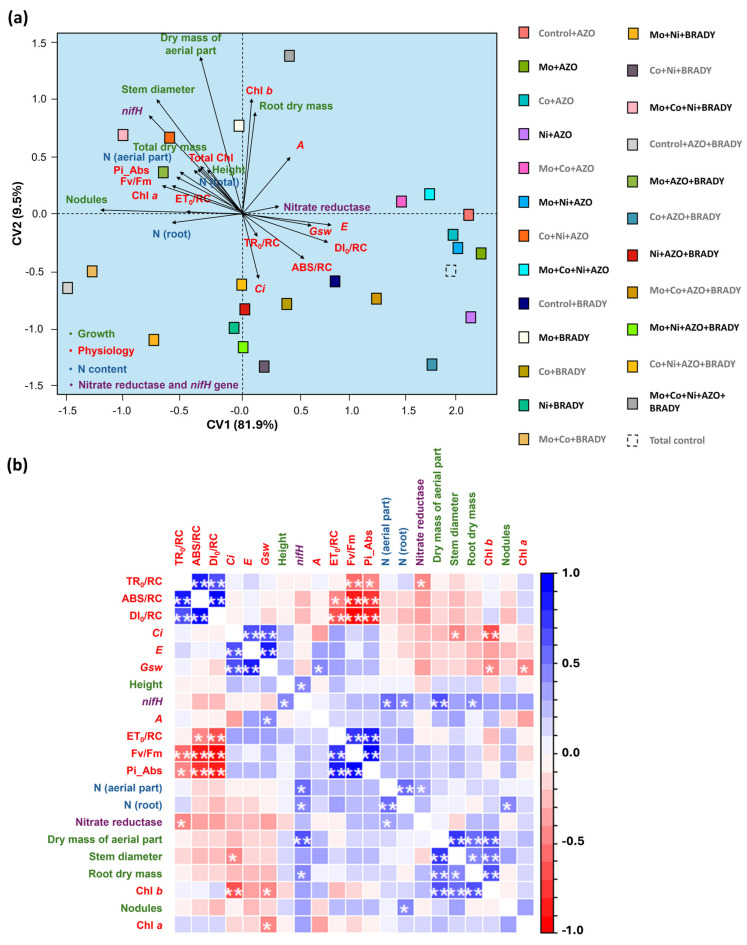
Canonical variable analysis (**a**) and correlation (**b**) of growth, physiological parameters (photosynthetic pigments, chlorophyll *a* fluorescence, and gas exchange), nitrogen content, nitrate reductase activity, and relative expression of the *nifH* gene in *Glycine max* L. plants subjected to different combinations of the micronutrients Mo, Co, and Ni and the diazotrophic rhizobacteria *Azospirillum brasilense* (AZO) and *Bradyrhizobium diazoefficiens* (BRADY). Chl *a* = chlorophyll *a*, Chl *b* = chlorophyll *b*, Total Chl = total chlorophyll. Asterisks indicate level of statistical significance: * *p* < 0.05, and ** *p* < 0.01.

The canonical axes explained 91.5% of the total variation in the dataset. Canonical variate analysis revealed that the complete treatment had a positive influence on *nifH* gene expression, stem diameter, shoot dry mass, root dry mass, chlorophyll *b* index, carbon assimilation rate (*A*), and total leaf N content (Figure 7a). In contrast, this treatment negatively influenced the ABS/RC and DI_0_/RC parameters, which were associated with higher Fv/Fm values, indicating improved photochemical efficiency.

Pearson’s correlation analysis showed a positive association between *nifH* gene expression and several growth parameters, including plant height, shoot dry mass, and root dry mass. As expected, *nifH* expression was also positively correlated with both shoot and root N concentrations (Figure 7b).

## 4. Discussion

### 4.1. The Combined Application of the Micronutrients Mo, Co, and Ni, Together with the Co-Inoculation of A. brasilense and B. diazoefficiens, Enhances the Vegetative Growth of G. max

Studies indicate that co-inoculation of *B. diazoefficiens* and *A. brasilense* enhances symbiotic efficiency in biological nitrogen fixation (BNF), and the agronomic benefits of this interaction have been validated on a large scale by farmers worldwide [7]. This synergistic effect is attributed to the ecological compatibility of these species [3,45,46] and their occupation of complementary niches. *B. diazoefficiens* is highly efficient in root nodule formation and atmospheric nitrogen fixation [47], whereas *A. brasilense* promotes root development through the production of phytohormones, particularly indole-3-acetic acid (IAA), thereby expanding the plant’s nutrient and water uptake capacity [48,49]. This synergistic interaction enhances BNF, likely improving nitrogen assimilation by plant tissues and resulting in higher accumulated N, especially in the roots [50].

Our findings corroborate those of Chibeba et al. [51], who reported that co-inoculation of *A. brasilense* and *Bradyrhizobium* spp. increased both shoot and root biomass in soybean plants compared to inoculation with *Bradyrhizobium* spp. alone. Similarly, Bazzo et al. [52] observed that co-inoculation of *G. max* with *B. japonicum* and *A. brasilense* improved vegetative growth parameters, including shoot dry mass, root biomass, and root length. This growth promotion is attributed to the functional complementarity of the two microorganisms: *A. brasilense* produces phytohormones such as auxins, cytokinins, and gibberellins, which directly stimulate root development, while *Bradyrhizobium* expresses genes involved in nodulation [53,54,55].

Root growth is accompanied by the exudation of flavonoids that activate the *Bradyrhizobium nodD* gene. This, in turn, stimulates the production of Nod factors, which are molecular signals that induce morphological changes in plant roots, such as nodule formation and root hair curling—processes essential for bacterial colonization [56]. The synergistic interaction between *Bradyrhizobium* and *Azospirillum* promotes earlier and more effective nodulation [57] while also enhancing nitrogenase activity in the nodules, thereby increasing nitrogen supply to the plant. Consequently, this leads to improved growth, productivity, and nitrogen use efficiency, making co-inoculation an agronomically advantageous and environmentally sustainable practice [58].

Co-inoculation, combined with supplementation of Mo, Co, and Ni, ensured high efficiency of BNF. Mo availability is critical for BNF because it functions as a cofactor for the molybdoenzyme nitrogenase, which catalyzes the conversion of atmospheric nitrogen (N_2_) into ammonia (NH_3_), the assimilable form of nitrogen for plants [21,26,59,60]. Molybdenum (Mo) plays a significant role in the formation of the metalloenzyme mARC (mitochondrial amidoxime-reducing component), and studies suggest that mARC may be involved in nitric oxide (NO) homeostasis. This occurs either through the reduction of NO precursors [61] or by acting as a nitrite-dependent nitric oxide synthase, meaning it catalyzes a one-electron reduction of nitrite to NO. This latter function is particularly relevant for nitrogen metabolism [62].

For the genus *Rhizobium*, NO production occurs during the symbiosis with legumes, spanning from the initial interaction through to the formation of nitrogen-fixing nodules [63,64]. Signorelli et al. [65] investigated the role of NO in this legume–rhizobia symbiosis and inferred that, although this reactive species can potentially reduce nitrogenase activity, NO exerts positive effects on BNF. Notably, the negative effects of NO require direct interaction with nitrogenase, while the positive effects are linked to signaling functions that can amplify beneficial processes.

Similarly, Co, though required in trace amounts, is essential for BNF as it is a constituent of cobalamin [15,66]. In *Rhizobium* and *Bradyrhizobium* species, cobalamin-dependent enzymes such as methionine synthase, methylmalonyl-CoA mutase, and ribonucleotide reductase play key roles in nodulation and nitrogen fixation [28]. Co deficiency significantly impairs methionine synthase activity, reducing methionine biosynthesis, which subsequently limits protein synthesis and results in smaller bacteroids [67]. Similarly, methylmalonyl-CoA mutase catalyzes the production of leghemoglobin, and Co limitation directly reduces leghemoglobin synthesis, thereby decreasing nitrogen fixation and limiting N supply to the plant. Leghemoglobin functions to protect nitrogenase from oxygen, so its shortage compromises nitrogenase activity [68]. Ribonucleotide reductase catalyzes the reduction of ribonucleotides to deoxyribonucleotides, a rate-limiting step in DNA synthesis and, consequently, in cell division and growth [69]. Ni, in turn, activates urease in leaves, an enzyme responsible for metabolizing nitrogenous compounds such as ureides, which, upon breakdown, maximize nitrogen assimilation in soybean plants [70,71].

The Co + Ni + BRADY and Co + AZO + BRADY treatments, however, reduced shoot dry mass and total dry mass of *G. max*. Although micronutrient supplementation in combination with rhizobacteria can enhance legume growth, excessive Co can disrupt symbiosis, impair BNF, and cause biomass losses. Barbosa et al. [11] reported that co-inoculation of *Bradyrhizobium* and *Azospirillum*, combined with high doses of Co and Mo, decreased BNF efficiency and compromised plant development. Other studies indicate that elevated Co concentrations interfere with bacteroid differentiation and functionality within root nodules, reducing nitrogen-fixing capacity while generating oxidative stress, reducing metabolic efficiency, and ultimately limiting plant growth [26,72].

High doses of micronutrients such as Mo, Co and Ni are potentially toxic to soybean crops due to multiple mechanisms. These elements induce oxidative stress and damage membranes and proteins [73]. Additionally, high concentrations of these metals can result in nutritional antagonism, blocking the absorption and transport of other essential nutrients [26]. Specifically in soybeans, an excess of these micronutrients in the sowing zone is phytotoxic to the seedling [74] and, crucially, to *Bradyrhizobium* in the symbiotic system, impairing inoculant survival and inducing evident epigenetic toxicity in the plants [75], which severely affects the crop’s agronomic and physiological parameters. Conversely, it is also important to note that these micronutrients, if applied in high quantities to the soybean crop, can be translocated to the grains and subsequently disseminated to higher levels of the food chain, posing a risk of toxicity to both animals and humans [76,77].

### 4.2. The Combined Application of the Micronutrients Mo, Co, and Ni, Together with Co-Inoculation of A. brasilense and B. diazoefficiens, Enhances the Primary Photochemical Performance of G. max

The Mo + Co + Ni + AZO + BRADY combination promoted higher efficiency values for Fv/Fm, ET_0_/RC, and PI_ABS while reducing photochemical stress parameters (ABS/RC and DI_0_/RC), suggesting that this treatment optimizes the use of absorbed light energy, mitigates stress in PSII, and enhances energy availability for plant growth and development. Oliveira et al. [78] demonstrated that Mo improves photosynthesis and nitrogen metabolism in soybean and maize, enhancing PSII efficiency and nitrogen assimilation. Adequate Co levels also play an antioxidant role, reducing the accumulation of reactive oxygen species (ROS) [28,79], which protects PSII from oxidative damage and enables optimized electron transport, as reflected by higher ET_0_/RC values in plants subjected to the complete treatment [80]. The observed decrease in DI_0_/RC further corroborates this protection, indicating reduced energy dissipation as heat, which is critical for maximizing the use of light energy [81,82].

The complete treatment also stimulated the accumulation of chlorophyll *b* in the leaves of *G. max*. Chlorophyll *b* complements chlorophyll *a* by aiding in light absorption [83]. This increase may be linked to greater nitrogen availability due to more efficient nitrogen fixation by bacteroids under the complete treatment, which favors the synthesis of photosynthetic pigments and PSII-associated proteins [84,85]. Similarly, in gas exchange measurements, the Mo + Co + Ni + AZO + BRADY combination resulted in significantly higher CO_2_ assimilation rates compared to the control, reflecting improved photosynthetic efficiency supported by enhanced light capture via chlorophyll *b* [86,87].

### 4.3. Mo, Co, and Ni Optimize Co-Inoculation, Increasing the Expression of the Gene nifH and BNF in Soybean Nodules

Nitrogen (N) is essential for plant growth and energy metabolism, being a constituent of key macromolecules such as amino acids, proteins, chlorophylls, and nucleic acids [88]. In soybean plants subjected to the complete treatment, supplementation with essential micronutrients (Mo, Co, and Ni) was crucial for maximizing nitrogen metabolism efficiency. Molybdenum and iron are essential for nitrogenase functionality, with Mo forming part of the active site responsible for reducing N_2_ to NH_3_ [89]. Nickel acts as a cofactor for urease, facilitating efficient N recycling in leaves; its deficiency impairs nitrogen assimilation and can indirectly reduce BNF via a “feedback effect” [90]. Cobalt (Co), in turn, supports nitrogenase activity and regulates energy metabolism in symbiotic microorganisms. It acts by promoting leghemoglobin synthesis, thereby protecting the nitrogenase enzyme against oxygen inactivation [91].

Plants inoculated solely with *A. brasilense* generally exhibited lower tissue N levels, as expected based on the bacterium’s mechanism of action. While *B. diazoefficiens* fixes atmospheric N directly via nitrogenase within root nodules [47], *A. brasilense* acts indirectly, stimulating root growth through phytohormone production and contributing to BNF in the rhizosphere [92,93].

The combined Mo + Co + Ni + AZO + BRADY treatment enhanced *nifH* gene expression in soybean nodules, with expression positively correlated with plant height, shoot dry mass, and root dry mass. The *nifH* gene encodes the dinitrogenase reductase subunit, a central component of the nitrogenase complex, and is widely used as a molecular marker for BNF [94,95]. Thus, an adequate supply of essential micronutrients, together with the synergistic interaction between *A. brasilense* and *B. diazoefficiens*, establishes optimal biochemical conditions for *nifH* induction and maintenance, enhancing BNF efficiency, improving physiological processes, and positively affecting plant growth and productivity.

## 5. Conclusions

Our results confirm the hypothesis that the combined application of the micronutrients Mo + Co + Ni, together with the co-inoculation of *A. brasilense* and *B. diazoefficiens*, significantly enhances vegetative growth, photochemical efficiency, and BNF in soybean plants. This treatment stimulated *nifH* gene expression, a key marker of BNF, resulting in improved N assimilation and enhanced photosynthetic performance, including increased PSII efficiency and net carbon assimilation (*A*). However, the data also indicate that excessive application of certain micronutrients, particularly Co, can impair symbiosis and reduce plant growth, underscoring the importance of proper dose management. These findings highlight the agronomic potential of integrating co-inoculation with targeted mineral supplementation as a strategy for sustainable soybean production. To promote environmentally responsible cultivation of *G. max*, further research is warranted to optimize micronutrient doses and combinations, evaluate their effects across diverse soils, climates, and soybean genotypes, and ultimately maximize BNF efficiency.

## Figures and Tables

**Table 1:** Physicochemical properties of the dystroferric red latosol used for cultivating *Glycine max* L. under treatments combining cobalt sulfate (Co), ammonium molybdate (Mo), and nickel sulfate (Ni) with the diazotrophic rhizobacteria *Azospirillum brasilense* (AZO) and *Bradyrhizobium diazoefficiens* (BRADY).

Ca	Mg	Ca^+^Mg	Al	H^+^Al	K	K	S	P	CaCl_2_
cmol_c_ dm^−3^						mg dm^−3^			pH
1.57	0.90	2.47	0.06	2.22	0.34	133	11.4	23.81	5.0
**Na**	**Fe**	**Mn**	**Cu**	**Zn**	**B**	**CEC**	**SB**	**V**	**m**
mg dm^−3^						cmol_c_ dm^−3^		%	
0.0	31.3	154.5	4.00	1.18	0.10	5.03	2.81	55	2.1
**Texture (g kg^−1^)**			**OM**	**Ca/Mg**	**Ca/K**	**Mg/K**	**Ca/CEC**	**Mg/CEC**	**K/CEC**
Clay	Silt	Sand	g dm^−3^	Relationship between bases					
48	8	44	36.1	1.7	4.6	2.7	31.21	17.89	6.76

P (phosphorus)—Mehlich 1, K (potassium), Na (sodium), Cu (copper), Fe (iron), Mn (manganese) and Zn (zinc)—Melich 1; Ca (calcium), Mg (magnesium) and Al (aluminum)—KCl 1 mol L^−1^; S (sulfur)—Ca (H_2_PO_4_)_2_ 0.01 mol L^−1^; OM (organic matter)—colorimetric method; B (boron)—hot water; CEC—cation exchange capacity; SB—sum of bases; V%—base saturation; and m%—aluminum saturation.

**Table 2:** Formulations combining ammonium molybdate (Mo), cobalt sulfate (Co), and nickel sulfate (Ni) with the diazotrophic rhizobacteria *Azospirillum brasilense* (AZO) and *Bradyrhizobium diazoefficiens* (BRADY) for the nutrition and inoculation of *Glycine max* L. plants.

Treatment	Formulations
Control + AZO	*A. brasilense*
Mo + AZO	30 g ha^−1^ ammonium molybdate + *A. brasilense*
Co + AZO	3 g ha^−1^ cobalt sulfate + *A. brasilense*
Ni + AZO	30 g ha^−1^ nickel sulfate + *A. brasilense*
Mo + Co + AZO	30 g ha^−1^ ammonium molybdate + 3 g ha^−1^ cobalt sulfate + *A. brasilense*
Mo + Ni + AZO	30 g ha^−1^ ammonium molybdate + 30 g ha^−1^ nickel sulfate + *A. brasilense*
Co + Ni + AZO	3 g ha^−1^ cobalt sulfate + 30 g ha^−1^ nickel sulfate + *A. brasilense*
Mo + Co + Ni + AZO	30 g ha^−1^ ammonium molybdate + 3 g ha^−1^ cobalt sulfate + 30 g ha^−1^ nickel sulfate + *A. brasilense*
Control + BRADY	*B. diazoefficiens*
Mo + BRADY	30 g ha^−1^ ammonium molybdate + *B. diazoefficiens*
Co + BRADY	3 g ha^−1^ cobalt sulfate + *B. diazoefficiens*
Ni + BRADY	30 g ha^−1^ nickel sulfate + *B. diazoefficiens*
Mo + Co + BRADY	30 g ha^−1^ ammonium molybdate + 3 g ha^−1^ cobalt sulfate + *B. diazoefficiens*
Mo + Ni + BRADY	30 g ha^−1^ ammonium molybdate + 30 g ha^−1^ nickel sulfate + *B. diazoefficiens*
Co + Ni + BRADY	3 g ha^−1^ cobalt sulfate + 30 g ha^−1^ nickel sulfate + *B. diazoefficiens*
Mo + Co + Ni + BRADY	30 g ha^−1^ ammonium molybdate + 3 g ha^−1^ cobalt sulfate + 30 g ha^−1^ nickel sulfate + *B. diazoefficiens*
Control + AZO + BRADY	*A. brasilense* + *B. diazoefficiens*
Mo + AZO + BRADY	30 g ha^−1^ ammonium molybdate + *A. brasilense* + *B. diazoefficiens*
Co + AZO + BRADY	3 g ha^−1^ cobalt sulfate + *A. brasilense* + *B. diazoefficiens*
Ni + AZO + BRADY	30 g ha^−1^ nickel sulfate + *A. brasilense* + *B. diazoefficiens*
Mo + Co + AZO + BRADY	30 g ha^−1^ ammonium molybdate + 3 g ha^−1^ cobalt sulfate + *A. brasilense* + *B. diazoefficiens*
Mo + Ni + AZO + BRADY	30 g ha^−1^ ammonium molybdate + 30 g ha^−1^ nickel sulfate + *A. brasilense* + *B. diazoefficiens*
Co + Ni + AZO + BRADY	3 g ha^−1^ cobalt sulfate + 30 g ha^−1^ nickel sulfate + *A. brasilense* + *B. diazoefficiens*
Mo + Co + Ni + AZO + BRADY	30 g ha^−1^ ammonium molybdate + 3 g ha^−1^ cobalt sulfate + 30 g ha^−1^ nickel sulfate + *A. brasilense* + *B. diazoefficiens*
Total control	Untreated seeds (lack of micronutrients and rhizobacteria)

## Data Availability

All the data relevant to this manuscript are available on request from the corresponding author.

## References

[1] Soumare A., Diedhiou A.G., Thuita M., Hafidi M., Ouhdouch Y., Gopalakrishnan S., Kouisni L. (2020). Exploiting biological nitrogen fixation: A route towards a sustainable agriculture. Plants.

[2] Zilli J.É., Alves B.J.R., Rouws L.F.M., Simões-Araujo J.L., de Barros Soares L.H., Cassán F., Castellanos M.O., O’Hara G. (2020). The importance of denitrification performed by nitrogen-fixing bacteria used as inoculants in South America. Plant Soil.

[3] Hungria M., Nogueira M.A., Araujo R.S. (2015). Soybean seed co-inoculation with *Bradyrhizobium* spp. and *Azospirillum brasilense*: A new biotechnological tool to improve yield and sustainability. Am. J. Plant Sci..

[4] Garcia M.V.C., Nogueira M.A., Hungria M. (2021). Combining microorganisms in inoculants is agronomically important but industrially challenging: Case study of a composite inoculant containing *Bradyrhizobium* and *Azospirillum* for the soybean crop. AMB Expr..

[5] Marks B.B., Megías M., Nogueira M.A., Hungria M. (2013). Biotechnological potential of rhizobial metabolites to enhance the performance of *Bradyrhizobium* spp. and *Azospirillum brasilense* inoculants with soybean and maize. AMB Expr..

[6] Rondina A.B.L., dos Santos Sanzovo A.W., Guimarães G.S., Wendling J.R., Nogueira M.A., Hungria M. (2020). Changes in root morphological traits in soybean co-inoculated with *Bradyrhizobium* spp. and *Azospirillum brasilense* or treated with *A. brasilense* exudates. Biol. Fertil. Soils.

[7] Prando A.M., Barbosa J.Z., de Oliveira A.B., Nogueira M.A., Possamai E.J., Hungria M. (2024). Benefits of soybean co-inoculation with *Bradyrhizobium* spp. and *Azospirillum brasilense*: Large-scale validation with farmers in Brazil. Eur. J. Agron..

[8] Figueiredo M.V.B., Mergulhão A.C.E.S., Sobral J.K., Lira Junior M.A., Araújo A.S.F., Arora N. (2013). Biological nitrogen fixation: Importance, associated diversity, and estimates. Plant Microbe Symbiosis: Fundamentals and Advances.

[9] Saha B., Saha S., Das A., Bhattacharyya P.K., Basak N., Sinha A.K., Poddar P., Meena V., Mishra P., Bisht J., Pattanayak A. (2017). Biological nitrogen fixation for sustainable agriculture. Agriculturally Important Microbes for Sustainable Agriculture: Volume 2: Applications in Crop Production and Protection.

[10] Fachinelli R., Ceccon G. (2020). *Bradyrhizobium* and *Azospirillum* coinoculation in soybean in succession to safrinha corn in sandy and clay soil. Acta Iguazu.

[11] Barbosa H.M., Alvarez R.D.C.F., Lima S.F.D., Cordeiro M.A.S., Zanella M.S., Bernardo V.F. (2022). *Bradyrhizobium* and *Azospirillum* co-inoculation associated with cobalt and molybdenum application in the soybean crop. Ciênc. Rural.

[12] Bonilla I., Bolaños L., Lichtfouse E. (2009). Mineral nutrition for legume–rhizobia symbiosis: B, Ca, N, P, S, K, Fe, Mo, Co, and Ni: A review. Organic Farming, Pest Control and Remediation of Soil Pollutants. Sustainable Agriculture Reviews.

[13] Weisany W., Raei Y., Allahverdipoor K.H. (2013). Role of some mineral nutrients in biological nitrogen fixation. Bull. Environ. Pharmacol. Life Sci..

[14] Lima E.G., Zanuzo M.R., Vieira C.V., Pelissari F., de Andrade Coimbra R. (2024). Effect of nickel seed treatment in soybeans. Sci. Electron. Arch..

[15] Hu X., Wei X., Ling J., Chen J., Pandey S., Tripathi D.K., Singh V.P., Sharma S., Chauhan D.K. (2023). Cobalt in plant life: Responses and deficiency symptoms. Beneficial Chemical Elements of Plants: Recent Developments and Future Prospects.

[16] Fatima P., Mishra A., Om H., Saha B., Kumar P., Kaushik B.D., Kumar D., Shamim M. (2019). Free living nitrogen fixation and their response to agricultural crops. Biofertilizers and Biopesticides in Sustainable Agriculture.

[17] Verma D.K., Kaur B., Pandey A.K., Asthir B., Verma D.K. (2019). Nitrogenase: A key enzyme in microbial nitrogen fixation for soil health. Microbiology for Sustainable Agriculture, Soil Health, and Environmental Protection.

[18] Fenice M., Gonzalez-Lopez J., Gonzalez-Martinez A. (2021). The nitrogen cycle: An overview. Nitrogen Cycle.

[19] Lavres J., Castro Franco G., de Sousa Câmara G.M. (2016). Soybean seed treatment with nickel improves biological nitrogen fixation and urease activity. Front. Environ. Sci..

[20] Izaguirre-Mayoral M.L., Lazarovits G., Baral B. (2018). Ureide metabolism in plant-associated bacteria: Purine plant-bacteria interactive scenarios under nitrogen deficiency. Plant Soil.

[21] Kumari R., Bhatnagar S., Kalra C., Yousuf P.Y., Shabir P.A., Hakeem K.R. (2022). Nitrogen assimilation in plants. Advances in Plant Nitrogen Metabolism.

[22] Lal M.A., Bhatla S.C., Bhatla S.C., Lal M.A. (2023). Nitrogen metabolism. Plant Physiology, Development and Metabolism.

[23] Matiz A., Mioto P.T., Mercier H., Cánovas F., Lüttge U., Leuschner C., Risueño M.C. (2019). Urea in plants: Metabolic aspects and ecological implications. Progress in Botany.

[24] Ono Y., Fukasawa M., Sueyoshi K., Ohtake N., Sato T., Tanabata S., Toyota R., Higuchi K., Saito A., Ohyama T. (2021). Application of nitrate, ammonium, or urea changes the concentrations of ureides, urea, amino acids and other metabolites in xylem sap and in the organs of soybean plants (*Glycine max* (L.) Merr.). Int. J. Mol. Sci..

[25] Rana M., Sankhyan N.K., Thakur P., Babal B., Anjali, Sharma S., Kumari S., Kumar P. (2025). Molybdenum in soil-plant system: Bioavailability, dynamics and implications for sustainable crop production. Discov. Soil.

[26] Roychoudhury A., Chakraborty S., Kumar V., Srivastava A.K., Suprasanna P. (2022). Cobalt and molybdenum: Deficiency, toxicity, and nutritional role in plant growth and development. Plant Nutrition and Food Security in the Era of Climate Change.

[27] Huang X.Y., Hu D.W., Zhao F.J. (2022). Molybdenum: More than an essential element. J. Exp. Bot..

[28] Hu X., Wei X., Ling J., Chen J. (2021). Cobalt: An essential micronutrient for plant growth?. Front. Plant Sci..

[29] Rabinovich A., Di R., Lindert S., Heckman J. (2024). Nickel and soil fertility: Review of benefits to environment and food security. Environments.

[30] Dilawari R., Kaur N., Priyadarshi N., Prakash I., Patra A., Mehta S., Singh B., Jain P., Islam M.A., Wani S.H., ul Rehman Sofi N., Bhat M.A., Lin F. (2022). Soybean: A key player for global food security. Soybean Improvement: Physiological, Molecular and Genetic Perspectives.

[31] Islam M.S., Muhyidiyn I., Islam M.R., Hasan M.K., Hafeez A.G., Hosen M.M., Saneoka H., Ueda A., Liu L., Naz M., Ohyama T., Takahashi Y., Ohtake N., Sato T., Tanabata S. (2022). Soybean and sustainable agriculture for food security. Soybean—Recent Advances in Research and Applications.

[32] Kumari S., Dambale A.S., Samantara R., Jincy M., Bains G., Singh K.P., Singh N.K., Aravind T. (2025). Introduction, history, geographical distribution, importance, and uses of soybean (*Glycine max* L.). Soybean Production Technology.

[33] Bhangu R., Virk H.K. (2019). Nitrogen management in soybean: A review. Agric. Rev..

[34] Almeida L.H.C., Nadur M.A., de Almeida P.P.S., Carrilho E.M., Ventura M.U., de Freitas Fregonezi G.A. (2025). Cell protector, cobalt, and molybdenum in association with *Bradyrhizobium* and *Azospirillum* in soybean cultivation. Obs. Econ. Latinoam..

[35] Silveira P.G., da Silva E.A.R., Nakao A.H., de Carvalho J.B. (2021). Efeito de doses de cobalto e molibdênio aplicadas no sulco de plantio da soja inoculada com *Bradyrhizobium*. Unifunec Científica Multidiscip..

[36] The Sociedade Brasileira de Ciência do Solo (SBCS) (2016). Manual de Calagem e Adubação para os Estados do Rio Grande do Sul e de Santa Catarina.

[37] Picazevicz A.A., Kusdra J.F., Moreno A.D.L. (2017). Maize growth in response to *Azospirillum brasilense*, *Rhizobium tropici*, molybdenum and nitrogen. Rev. Bras. Eng. Agríc. Ambient..

[38] Marcondes J.A.P., Caires E.F. (2005). Aplicação de molibdênio e cobalto na semente para cultivo da soja. Bragantia.

[39] Strasser R.J., Srivastava A., Tsimilli-Michael M., Yunus M., Pathre U., Mohanty P. (2000). The fluorescence transient as a tool to characterize and screen photosynthetic samples. Probing Photosynthesis: Mechanisms, Regulation and Adaptation.

[40] Malavolta E., Vitti G.C., Oliveira S.A. (1997). Avaliação do Estado Nutricional das Plantas: Princípios e Aplicações.

[41] Hageman R.H., Reed A.J., Olshansky L. (1980). Nitrate reductase from higher plants. Methods in Enzymology.

[42] Azevedo A.M. Package ‘MultivariateAnalysis’. https://cran.r-project.org/web/packages/MultivariateAnalysis/index.html.

[43] Wei T., Simko V. R Package ‘Corrplot’: Visualization of a Correlation Matrix. https://github.com/taiyun/corrplot.

[44] R Core Team (2025). R: A Language and Environment for Statistical Computing.

[45] Torres D., Donadio F., López G., Molina R., Obando M., Nievas S., Rosas S., Ćavar Zeljković S., Díaz-Zorita M., De Diego N. (2022). Previous incubation of *Bradyrhizobium japonicum* E109 and *Azospirillum argentinense* Az39 (formerly *A. brasilense* Az39) improves the *Bradyrhizobium*–soybean symbiosis. J. Soil Sci. Plant Nutr..

[46] Martin T.N., Vey R.T., Vieira F.C.B., Jacques R.J.S., Ferreira M.M. (2023). How did the coinoculation of *Bradyrhizobium* and *Azospirillum* become indispensable for soybean production in Brazil?. Symbiosis.

[47] Nicoud Q., Lamouche F., Chaumeret A., Balliau T., Le Bars R., Bourge M., Pierre F., Guérard F., Sallet E., Tuffigo S. (2021). *Bradyrhizobium diazoefficiens* USDA110 nodulation of *Aeschynomene afraspera* is associated with atypical terminal bacteroid differentiation and suboptimal symbiotic efficiency. mSystems.

[48] Coniglio A., Mora V., Puente M., Cassán F., Zúñiga-Dávila D., González-Andrés F., Ormeño-Orrillo E. (2019). *Azospirillum* as biofertilizer for sustainable agriculture: *Azospirillum brasilense* AZ39 as a model of PGPR and field traceability. Microbial Probiotics for Agricultural Systems: Advances in Agronomic Use.

[49] Fukami J., Cerezini P., Hungria M. (2018). *Azospirillum*: Benefits that go far beyond biological nitrogen fixation. AMB Expr..

[50] Santos M.S., Nogueira M.A., Hungria M. (2021). Outstanding impact of *Azospirillum brasilense* strains Ab-V5 and Ab-V6 on the Brazilian agriculture: Lessons that farmers are receptive to adopt new microbial inoculants. Rev. Bras. Ciênc. Solo.

[51] Chibeba A.M., Guimarães M.F., Brito O.R., Nogueira M.A., Araújo R.S., Hungria M. (2015). Co-inoculação de soja com *Bradyrhizobium* e *Azospirillum* promove nodulação precoce. Am. J. Plant Sci..

[52] Bazzo J.H.B., Monteiro J., de Lucena Marinho J. (2020). Inoculação e coinoculação de *Azospirillum* e *Bradyrhizobium*, via sementes e em cobertura, na qualidade fisiológica de sementes de soja. Rev. Cult. Agron..

[53] Yuan S., Zhou S., Feng Y., Zhang C., Huang Y., Shan Z., Chen S., Guo W., Yang H., Yang Z. (2021). Identification of the important genes of *Bradyrhizobium diazoefficiens* 113-2 involved in soybean nodule development and senescence. Front. Microbiol..

[54] Zaheer M.S., Ali H.H., Iqbal M.A., Erinle K.O., Javed T., Iqbal J., Makhdoom I.U.H., Mumtaz M.Z., Salama E.A.A., Kalaji H.M. (2022). Cytokinin production by *Azospirillum brasilense* contributes to increase in growth, yield, antioxidant, and physiological systems of wheat (*Triticum aestivum* L.). Front. Microbiol..

[55] Ganusova E.E., Banerjee I., Seats T., Alexandre G. (2025). Indole-3-acetic acid (IAA) protects *Azospirillum brasilense* from indole-induced stress. Appl. Environ. Microbiol..

[56] Fernandez-Göbel T.F., Deanna R., Muñoz N.B., Robert G., Asurmendi S., Lascano R. (2019). Redox systemic signaling and induced tolerance responses during soybean–*Bradyrhizobium japonicum* interaction: Involvement of nod factor receptor and autoregulation of nodulation. Front. Plant Sci..

[57] Zeffa D.M., Fantin L.H., Koltun A., de Oliveira A.L.M., Nunes M.P.B.A., Canteri M.G., Gonçalves L.S.A. (2020). Effects of plant growth-promoting rhizobacteria on co-inoculation with *Bradyrhizobium* in soybean crop: A meta-analysis of studies from 1987 to 2018. PeerJ.

[58] Chibeba A.M., Kyei-Boahen S., de Fátima Guimarães M., Nogueira M.A., Hungria M. (2020). Towards sustainable yield improvement: Field inoculation of soybean with *Bradyrhizobium* and co-inoculation with *Azospirillum* in Mozambique. Arch. Microbiol..

[59] Pahari A., Nayak S.K., Banik A., Lakra P.B., Mishra B.B., Mishra B.B., Nayak S.K., Pahari A. (2021). Biological nitrogen fixation mechanism and applications. Agriculturally Important Microorganisms.

[60] Devi O.R., Sarma A., Borah K., Prathibha R.S., Tamuly G., Maniratnam K., Laishram B. (2023). Importance of zinc and molybdenum for sustainable pulse production in India. Environ. Ecol..

[61] Kotthaus J., Kotthaus J., Schade D., Schwering U., Hungeling H., Müller-Fielitz H., Raasch W., Clement B. (2011). New prodrugs of the antiprotozoal drug pentamidine. ChemMedChem.

[62] Clement B., Struwe M.A. (2023). The history of mARC. Molecules.

[63] Oliveira M.S., Santos K.F., de Paula R.M., Vitorino L.C., Bessa L.A., Greer A., Di Mascio P., De Souza J.C.P., Martin-Didonet C.C. (2023). Nitric oxide detection using a chemical trap method for applications in bacterial systems. Microorganisms.

[64] Puppo A., Pauly N., Boscari A., Mandon K., Brouquisse R. (2013). Hydrogen peroxide and nitric oxide: Key regulators of the Legume-*Rhizobium* and mycorrhizal symbioses. Antioxid. Redox Signal..

[65] Signorelli S., Sainz M., Rosa S.T., Monza J. (2020). The role of nitric oxide in nitrogen fixation by legumes. Front. Plant Sci..

[66] Akeel A., Jahan A., Naeem M., Ansari A., Gill S. (2020). Role of cobalt in plants: Its stress and alleviation. Contaminants in Agriculture: Sources, Impacts and Management.

[67] Marschner P. (2011). Marschner’s Mineral Nutrition of Higher Plants.

[68] Hopkins W. (1995). Introduction to Plant Physiology.

[69] Kolberg M., Strand K.R., Graff P., Andersson K.K. (2004). Structure, function, and mechanism of ribonucleotide reductases. Biochim. Biophys. Acta (BBA)-Proteins Proteom..

[70] Gerendás J., Polacco J.C., Freyermuth S.K., Sattelmacher B. (1999). Significance of nickel for plant growth and metabolism. J. Plant Nutr. Soil Sci..

[71] Gupta S., Yildirim S., Andrikopoulos B., Wille U., Roessner U. (2023). Deciphering the interactions in the root–soil nexus caused by urease and nitrification inhibitors: A review. Agronomy.

[72] Salam A., Afridi M.S., Khan A.R., Azhar W., Shuaiqi Y., Ulhassan Z., Qi J., Xuo N., Chunyan Y., Chen N., Hossain M.A., Hossain A.K.M.Z., Bourgerie S., Fujita M., Dhankher O.P., Haris P. (2023). Cobalt induced toxicity and tolerance in plants: Insights from omics approaches. Heavy Metal Toxicity and Tolerance in Plants: A Biological, Omics, and Genetic Engineering Approach.

[73] Chrysargyris A., Höfte M., Tzortzakis N., Petropoulos S.A., Di Gioia F. (2022). Micronutrients: The borderline between their beneficial role and toxicity in plants. Front. Plant Sci..

[74] Teixeira G.C.M., Prado R.D.M., Rocha A.M.S., Silva J.L.F.D., Lata-Tenesaca L.F., Dias M.A.N. (2023). The adequate dose of Mo required for soybean seed treatment is low when associated with Cu, Mn, and Zn compared to its association with Co and Ni, although increasing the risk of toxicity. J. Plant Nutr..

[75] Yang J., Song Z., Ma J., Han H. (2020). Toxicity of molybdenum-based nanomaterials on the soybean–*rhizobia* symbiotic system: Implications for nutrition. ACS Appl. Nano Mater..

[76] Razzaque M.S., Wimalawansa S.J. (2025). Minerals and human health: From deficiency to toxicity. Nutrients.

[77] Aljohani A.R., Alharbi S., Althobaitri R.S., Aljohani R.S., Alnemari R.O., Atida H. (2023). Micronutrients toxicity from causes to adverse effects: A review. Int. J. Med. Dev. Ctries..

[78] Oliveira S.L., Crusciol C.A.C., Rodrigues V.A., Galeriani T.M., Portugal J.R., Bossolani J.W., Moretti L.G., Calonego J.C., Cantarella H. (2022). Molybdenum foliar fertilization improves photosynthetic metabolism and grain yields of field-grown soybean and maize. Front. Plant Sci..

[79] Ma J., Song Z., Yang J., Wang Y., Han H. (2021). Cobalt ferrite nanozyme for efficient symbiotic nitrogen fixation via regulating reactive oxygen metabolism. Environ. Sci. Nano.

[80] Tiwari S., Mchanty P. (1996). Cobalt induced changes in photosystem activity in *Synechocystis* PCC 6803: Alterations in energy distribution and stoichiometry. Photosynth. Res..

[81] El-Sheekh M.M., El-Naggar A.H., Osman M.E.H., El-Mazaly E. (2003). Effect of cobalt on growth, pigments and the photosynthetic electron transport in *Monoraphidium minutum* and *Nitzchia perminuta*. Braz. J. Plant Physiol..

[82] Krieger-Liszkay A., Shimakawa G. (2022). Regulation of the generation of reactive oxygen species during photosynthetic electron transport. Biochem. Soc. Trans..

[83] Kume A., Akitsu T., Nasahara K.N. (2018). Why is chlorophyll *b* only used in light-harvesting systems?. J. Plant Res..

[84] Zayed O., Hewedy O.A., Abdelmoteleb A., Ali M., Youssef M.S., Roumia A.F., Seymour D., Yuan Z.C. (2023). Nitrogen journey in plants: From uptake to metabolism, stress response, and microbe interaction. Biomolecules.

[85] Farhan M., Sathish M., Kiran R., Mushtaq A., Baazeem A., Hasnain A., Hakim F.F., Naqvi S.A.H., Mubeen M., Iftikhar Y. (2024). Plant nitrogen metabolism: Balancing resilience to nutritional stress and abiotic challenges. Phyton.

[86] Biswal A.K., Pattanayak G.K., Pandey S.S., Leelavathi S., Reddy V.S., Govindjee, Tripathy B.C. (2012). Light intensity-dependent modulation of chlorophyll *b* biosynthesis and photosynthesis by overexpression of chlorophyllide *a* oxygenase in tobacco. Plant Physiol..

[87] Miglani G.S., Kaur R., Sharma P., Gupta N. (2021). Leveraging photosynthetic efficiency toward improving crop yields. J. Crop Improv..

[88] Koch H., Sessitsch A. (2024). The microbial-driven nitrogen cycle and its relevance for plant nutrition. J. Exp. Bot..

[89] Cadoux C., Maslać N., Di Luzio L., Ratcliff D., Gu W., Wagner T., Milton R.D. (2023). The mononuclear metal-binding site of Mo-nitrogenase is not required for activity. JACS Au.

[90] Rodríguez-Jiménez T.D.J., Ojeda-Barrios D.L., Blanco-Macías F., Valdez-Cepeda R.D., Parra-Quezada R. (2016). Urease and nickel in plant physiology. Rev. Chapingo Ser. Hortic..

[91] Abreu I., Reguera M., Bonilla A., Bolaños L., Bonilla I., Rodelas González M.B., Gonzalez-Lopez J. (2016). Mineral nutrition in the legume–rhizobia nitrogen fixing symbiosis. Beneficial Plant-Microbial Interactions: Ecology and Applications.

[92] Barbosa J.Z., Hungria M., da Silva Sena J.V., Poggere G., dos Reis A.R., Corrêa R.S. (2021). Meta-analysis reveals benefits of co-inoculation of soybean with *Azospirillum brasilense* and *Bradyrhizobium* spp. in Brazil. Appl. Soil Ecol..

[93] Reis A.F.B., Rosso L.H.M., Adee E., Davidson D., Kovács P., Purcell L.C., Below F.E., Casteel S.N., Knott C., Kandel H. (2022). Seed inoculation with *Azospirillum brasilense* in the U.S. soybean systems. Field Crops Res..

[94] Naorem A., Tilgam J., Priyadarshini P., Tak Y., Bharati A., Patel A., Yousuf P.Y., Shabir P.A., Hakeem K.R. (2022). Nitrogenase enzyme complex: Functions, regulation, and biotechnological applications. Advances in Plant Nitrogen Metabolism.

[95] Nichio B.T.D.L., Chaves R.B.R., Pedrosa F.D.O., Raittz R.T. (2025). Exploring diazotrophic diversity: Unveiling Nif core distribution and evolutionary patterns in nitrogen-fixing organisms. BMC Genom..

